# Clinical characteristics and outcomes of acute eosinophilic pneumonia: A nationwide descriptive study^☆^

**DOI:** 10.1016/j.waojou.2025.101139

**Published:** 2025-11-15

**Authors:** Tomohiro Akaba, Yuya Kimura, Mitsuko Kondo, Hiroki Matsui, Kiyohide Fushimi, Etsuko Tagaya, Hideo Yasunaga

**Affiliations:** a Department of Respiratory Medicine, Tokyo Women's Medical University School of Medicine, Tokyo, Japan; b Department of Clinical Epidemiology and Health Economics, School of Public Health, The University of Tokyo, Tokyo, Japan; c Department of Health Services Research, Graduate School of Medicine, The University of Tokyo, Tokyo, Japan; d Department of Health Policy and Informatics, Institute of Science Tokyo Graduate School of Medicine, Tokyo, Japan

**Keywords:** Acute eosinophilic pneumonia, Hypoxia, Descriptive study

## Abstract

Acute eosinophilic pneumonia (AEP) is a rare lung disease marked by rapid respiratory failure and eosinophilic infiltration of the lungs. Despite its clinical importance, limited data exist on the characteristics and outcomes of AEP in the general population. We conducted a nationwide study using the Japanese Diagnosis Procedure Combination database from April 2020 to March 2023. Patients with AEP were identified based on diagnostic codes and bronchoscopy records, and we evaluated their clinical characteristics, treatments, and outcomes. A total of 328 patients were included. The median age was 46 years, and 63.7% were male. Hypoxia was present in 86.9% of patients at admission. Systemic corticosteroids were administered to 96% of patients, with 37.5% receiving pulse therapy. Mechanical ventilation was required in 4.9% of cases, and 7.3% of patients were admitted to intensive or high-care units. The median hospital stay was 11 days, and in-hospital mortality was 1.2%. Patients with hypoxia at admission had longer hospital stays and received higher doses of corticosteroids and empirical antibiotics (p < 0.001 for each). No disease-related readmissions were reported after discharge. These findings suggest that most AEP patients have favorable short-term outcomes, although those with hypoxia at admission may require more intensive treatment.

## Introduction

Acute eosinophilic pneumonia (AEP) is a rare and rapidly progressing lung disease characterized by sudden onset of respiratory symptoms and eosinophilic lung infiltration.^1^ Patients typically present with fever, dyspnea, and hypoxemia, alongside diffuse pulmonary infiltrates on imaging. The diagnosis is confirmed through bronchoalveolar lavage (BAL) and/or lung biopsy, which reveals eosinophil accumulation in the lung. Although AEP is often triggered by inhalational exposures such as cigarette smoke, dust, or chemical oxidants, some cases remain idiopathic.^2^ Since its first description in 1989, several case reports and small cohort studies have described its clinical features.^3^ However, large-scale epidemiological studies on AEP characteristics and outcomes remain limited.

## Methods

In this study, we conducted a retrospective analysis of AEP using data from the Japanese Diagnosis Procedure Combination (DPC) database, which includes inpatient data from over 1700 acute care hospitals between April 2020 and March 2023. The details of DPC have been described previously, and a validation study of the DPC database demonstrated high sensitivity and specificity for procedural records.^4^^,^^5^ In terms of diagnostic accuracy, most diagnostic records showed high specificity and moderate sensitivity.

Patients with AEP were identified based on the International Classification of Diseases, 10th Revision (ICD-10) code J82, and a documented primary diagnosis of “acute eosinophilic pneumonia.” To ensure diagnostic accuracy, only patients who underwent bronchoscopy were included, and those with malignancies or vasculitis, such as eosinophilic granulomatosis with polyangiitis, were excluded. Data on patient demographics, clinical characteristics, treatments, and outcomes were extracted. Given that previous studies have shown that the initial dose of systemic steroids is influenced by the presence of respiratory failure, we compared the clinical course and outcomes in patients with AEP based on the presence of hypoxia at admission.^6^ Hypoxia was defined as the need for oxygen on the first day of hospitalization. Additionally, we evaluated fatal AEP cases to identify potential risk factors associated with mortality in this otherwise generally favorable disease. Procedure and disease codes are listed in Supplementary Tables 1 and 2.

Continuous variables are presented as medians with interquartile ranges, while categorical variables are reported as frequencies and percentages. Fisher's exact test and the Wilcoxon rank sum test were used to compare categorical and continuous variables, respectively. Statistical significance was set at p < 0.05. All analyses were performed using Stata/SE 18.0 (Stata Corp., College Station, TX, USA). This study was approved by the institutional review board. All data were de-identified, and the requirement for written informed consent was waived.

## Results

During the study period, a total of 328 patients were included. The baseline characteristics are summarized in Table 1. The median age was 46 years (interquartile range, 21–66), and 63.7% were male. Hypoxia at admission was present in 86.9% of patients. AEP was most frequently observed in autumn. The clinical course and outcomes are detailed in Table 2. Systemic corticosteroids were administered to 96.0% of patients, with steroid pulse therapy used in 37.5%. In-hospital mortality was 1.2% (4/328), occurring only in patients with hypoxia at admission. No cases of disease-related readmission were reported. Patients with hypoxia at admission had significantly longer hospital stays and were more likely to receive high-dose corticosteroids and empirical antibiotics (p < 0.001). Detailed information on the fatal cases, including age and comorbidities, is presented in Supplementary Table 3. Among the 4 fatal cases, 2 deaths were attributed to AEP, while the other 2 were due to non-AEP causes (bacterial pneumonia and acute myocardial infarction).

**Table 1:** Baseline characteristics of eligible cases with AEP

	N = 328
Age (years)	46 (21–66)
Male	209 (63.7)
BMI (kg/m^2^)	
<18.50	37 (11.3)
18.50–24.99	201 (61.3)
25.00–29.99	49 (14.9)
≥30.00	10 (3.1)
Missing	31 (9.5)
Hugh Jones	
1, 2	187 (57.0)
3, 4	92 (28.1)
5	15 (4.6)
missing	34 (10.4)
Hypoxia at admission	285 (86.9)
Smoking index (pack-year)	
0	148 (45.1)
1–20	96 (29.3)
20–40	23 (7.0)
≥40	22 (6.7)
Missing data	39 (11.9)
Comorbidities	
COPD	5 (1.5)
Asthma	13 (4.0)
Allergic rhinitis	8 (2.4)
Chronic rhinosinusitis	5 (1.5)
Atopic dermatitis	2 (0.6)
Diabetes mellitus	9 (2.7)
Cardiovascular diseases	12 (3.7)
Connective tissue disease	5 (1.5)
Season at the presentation of AEP	
Spring (March–May)	74 (22.6)
Summer (June–August)	83 (25.3)
Autumn (September–November)	105 (32.0)
Winter (December–February)	66 (20.1)
Fiscal year	
2020	101 (30.8)
2021	120 (36.6)
2022	107 (32.6)

Continuous variables are presented as medians with interquartile ranges, and categorical variables as frequencies and percentages.

AEP, acute eosinophilic pneumonia.

**Table 2:** Clinical course and outcomes of all cases, cases with hypoxia, and cases without hypoxia

	Overall (N = 328)	AEP with hypoxia (N = 285)	AEP without hypoxia (N = 43)	p value
Length of hospital stay (day)	11 (8–16)	12 (9–17)	9 (5–13)	<0.001
Use of systemic corticosteroids	315 (96.0)	283 (99.3)	32 (74.4)	0.03
Initial dose of systemic corticosteroids^a^ (mg/day)	141 (57–535)	183 (60–675)	23 (15–75)	<0.001
Use of steroid pulse therapy^b^	123 (37.5)	114 (40)	9 (20.1)	0.02
Use of empirical antibiotics^c^	254 (77.4)	241 (84.6)	13 (30.2)	<0.001
Use of mechanical ventilation	16 (4.9)	15 (5.3)	1 (2.3)	0.71
Admission to HCU or ICU	24 (7.3)	24 (8.4)	0 (0)	0.06
Full ADL independence at discharge^d^	292 (89.0)	250 (87.2)	42 (97.7)	0.06
Discharge at home	320 (97.6)	277 (97.2)	43 (100)	0.60
Hospital death	4 (1.2)	4 (1.4)	0 (0)	1.00
Hospital readmission due to disease recurrence	0 (0)	0 (0)	0 (0)	1.00

Continuous variables are presented as medians with interquartile ranges, and categorical variables as frequencies and percentages.

ADL, activities of daily living; AEP, acute eosinophilic pneumonia; HCU, high care unit; ICU, intensive care unit.

a Prednisolone equivalent.

b Steroid pulse therapy refers to the administration of systemic steroids at doses ranging from 500 to 1000 mg/day as initial treatment.

c Except for sulfamethoxazole-trimethoprim, which is used to prevent *Pneumocystis* pneumonia.

d Full ADL independence at discharge is defined as a Barthel Index score of 100.

## Discussion

This study provides comprehensive data on AEP in the general population. Previous studies were limited by small sample sizes or focused on military personnel, which may not reflect broader populations.^7^^,^^8^ Our findings were partially consistent with earlier reports, though the median age was higher, and over 30% of patients were female. Although the exact cause of AEP remains unclear, it is frequently linked to smoking and environmental exposures. The higher prevalence in males may be related to smoking behaviors, though declining smoking rates in Japan could influence future demographics.^9^ As smoking rates decrease, other triggers, such as chemical oxidants or environmental factors, may become more prominent, affecting age and sex distribution. Comorbidities related to eosinophilic or allergic conditions were rare in AEP, reinforcing that AEP and chronic eosinophilic pneumonia (CEP) are distinct diseases. CEP, in contrast, frequently overlaps with conditions such as asthma, allergic rhinitis, and atopic dermatitis.^10^ The seasonal peak of AEP in autumn observed in our study may reflect the influence of environmental factors;^11^ nonetheless, this association should be interpreted cautiously. Other factors, including increased respiratory infections, seasonal health-care utilization changes, or incidental clustering, may further contribute to this pattern.

Most patients with AEP experienced favorable short-term outcomes during hospitalization, with a low mortality rate and no cases of disease recurrence or readmission. The database did not capture outpatient or long-term data; therefore, these results should be interpreted as reflecting short-term prognosis only. Rhee et al reported no fatal cases, whereas Shorr et al described 2 deaths among 18 patients.^6^^,^^7^ Here, in-hospital mortality was 1.2%, likely reflecting the inclusion of a broader and more heterogeneous population compared to that of earlier studies. The majority responded well to systemic corticosteroids, though some required steroid pulse therapy. Our findings regarding corticosteroid use were consistent with previous large studies.^6^^,^^7^ Notably, a few patients were discharged without corticosteroids, suggesting that mild cases may resolve spontaneously. In contrast, patients with hypoxia at admission required more intensive care, including higher steroid doses and admission to intensive or high-dependency care units, underscoring the importance of close monitoring in these cases. Finally, we did not evaluate the optimal corticosteroid dose or treatment duration; further research is warranted to establish appropriate regimens according to disease severity and baseline condition.

Our study has some limitations. As a database study, diagnostic accuracy depended on recorded ICD-10 codes and procedural data, and laboratory or radiologic findings were not available. Additionally, although the database included information on smoking index, detailed data on environmental or occupational exposures were unavailable. Therefore, potential AEP triggers could not be evaluated, and associations with smoking or exposure-related factors should be interpreted with caution. Moreover, because our case definition required bronchoscopy, patients diagnosed clinically without bronchoscopy were excluded, which may have introduced selection bias toward more severe cases. Although the retrospective design inherently limits control over diagnostic and therapeutic variability, the use of a standardized nationwide database and consistent case definitions may have minimized random bias across institutions. Moreover, misclassification may have occurred due to coding inaccuracies, and some variables, such as smoking status, contained missing data that could have affected the precision of our estimates. Despite these limitations, the large sample size offers valuable insights into AEP epidemiology and management. Future studies with access to detailed imaging and laboratory data could help clarify disease phenotypes. Furthermore, prospective evaluations of different corticosteroid regimens may guide the development of optimized treatment protocols.

In conclusion, this study, the largest to date, provides a comprehensive analysis of AEP characteristics, treatment patterns, and outcomes. While most patients had short-term favorable prognoses, those with hypoxia at admission required more intensive therapy. Further studies with long-term follow-up are warranted to clarify the overall prognosis and optimize AEP management strategies.

## Availability of data and materials

The data used in the present study are not publicly available owing to contracts with hospitals that provide data to the database.

## Author contributions

YK, HM, and KF contributed to data acquisition. TA, YK, MK, ET, and HY contributed to data analysis and drafted the manuscript. All authors contributed to the study design, interpreted the data, revised the manuscript, and approved the final manuscript.

## Ethics approval

This study was approved by the institutional review board of the University of Tokyo (approval number: 3501-(5)). All data were de-identified, and the requirement for written informed consent was waived.

## Disclosure of the use of Generative AI and AI-assisted Technologies

Nothing to disclose.

## Funding

This work was supported by grants from the Ministry of Health, Labor and Welfare, Japan (23AA2003 and 24AA2006).

## Declaration of competing interest

The authors have no actual or potential conflicts of interest to declare.

## Abbreviations

AEP, acute eosinophilic pneumonia; BAL, bronchoalveolar lavage; DPC, Diagnosis Procedure Combination; CEP, chronic eosinophilic pneumonia; ICD-10, International Classification of Diseases, 10th Revision.
