# Supplementary material for: Clinical characteristics and outcomes of acute eosinophilic pneumonia: A nationwide descriptive study
Source: World Allergy Organ J. 2025 Nov 15;18(11):101139. doi: 10.1016/j.waojou.2025.101139 (PMC12663830; doi:10.1016/j.waojou.2025.101139)
Supplement: Multimedia component 1 [file mmc1.docx]

**Supplementary data**

**Supplement Table 1**. List of bronchoscopy procedure codes

| **Procedure codes** |
| --- |
| D302 |
| D302-2 |
| D415 |
| D415-2 |
| D415-3 |
| D415-4 |
| D415-5 |
| D4151 |
| D4152 |

**Supplement Table 2.** ICD-10 codes for exclusion criteria and comorbidities

| **Diseases** | **ICD-10 codes** |
| --- | --- |
| ***Exclusion criteria*** |  |
| Malignancy | C00-C97, D00-D09 |
| Vasculitis | M30, M31 |
| ***Comorbidities*** |  |
| COPD | J43, J44, J324, J328, J329 |
| Asthma | J45, J46 |
| Allergic rhinitis | J301, J302, J303, J304 |
| Chronic sinusitis | J32 |
| Atopic dermatitis | L208, L209 |
| Diabetes mellitus | E10–E14 |
| Cardiovascular disease | I20–I25, I42, I50 |
| Connective tissue disease | M05–M09, M32-M36, K50–K51 |

ICD-10: International Classification of Diseases, Tenth Revision.

**Supplement Table 3.** Clinical characteristics of fatal cases of acute eosinophilic pneumonia

| **Case** | **Age (years)** | **Sex** | **Hypoxia at admission** | **ICU admission** | **Mechanical ventilation** | **Comorbidities** | **Initial steroid dose (mg/day)**^†^ | | **Steroid pulse therapy**^‡^ | **Antibiotics use** | **Length of stay (days)** | **Cause of death** |
| --- | --- | --- | --- | --- | --- | --- | --- | --- | --- | --- | --- | --- |
| 1 | 44 | Male | Yes | Yes | Yes | Bronchiectasis, Diabetes | | 1000 | Yes | Yes | 14 | Acute eosinophilic pneumonia |
| 2 | 57 | Male | Yes | No | No | Diabetes, Heart failure | | 70 | No | Yes | 10 | Bacterial pneumonia |
| 3 | 39 | Male | Yes | Yes | Yes | Diabetes, Hypertension | | 45 | No | Yes | 33 | Acute Myocardial infarction |
| 4 | 65 | Male | Yes | No | Yes | COPD, Diabetes | | 1000 | Yes | Yes | 25 | Acute eosinophilic pneumonia |

†Prednisolone equivalent.

‡Steroid pulse therapy refers to the administration of systemic steroids at doses ranging from 500 to 1000 mg/day as initial treatment.
